# Serum Myeloperoxidase and Nitric Oxide Levels in Acute Exacerbation and Stable COPD Compared with Healthy Controls: A Cross-Sectional Exploratory Biomarker Study

**DOI:** 10.3390/medicina62081558

**Published:** 2026-08-14

**Authors:** Gülşah Ethemoğlu, Nihayet Bayraktar, Hamza Erdoğdu, Hatice Kalaycıoğlu Avan

**Affiliations:** 1 Department of Chest Diseases, Faculty of Medicine, Harran University, Şanlıurfa 63200, Türkiye; 2 Department of Biochemistry, Faculty of Medicine, Harran University, Şanlıurfa 63200, Türkiye; 3 Department of Biostatistics, Faculty of Medicine, Harran University, Şanlıurfa 63200, Türkiye

**Keywords:** chronic obstructive pulmonary disease, acute exacerbation, myeloperoxidase, nitric oxide, oxidative stress, systemic inflammation

## Abstract

*Background and Objectives:* Oxidative stress and nitric oxide (NO)-related inflammatory activity contribute to chronic obstructive pulmonary disease (COPD), particularly during acute exacerbation of COPD (AECOPD). Myeloperoxidase (MPO) reflects neutrophil-related oxidative activity, whereas NO is involved in airway inflammation, endothelial regulation, and systemic inflammatory responses. This study compared serum MPO and NO levels among patients with AECOPD, patients with stable COPD, and healthy controls, and explored their discriminatory performance for distinguishing AECOPD from stable COPD. *Materials and Methods:* This cross-sectional comparative observational study prospectively enrolled 90 participants: 30 patients with AECOPD, 30 patients with stable COPD, and 30 healthy adult controls. COPD was confirmed by post-bronchodilator spirometry. Serum MPO and NO levels were measured using commercially available enzyme-linked immunosorbent assay kits. Group comparisons, exploratory subgroup analyses, correlation analyses, regression models, and receiver operating characteristic (ROC) curve analyses were performed. *Results:* Serum NO and MPO levels increased stepwise from healthy controls to stable COPD and AECOPD. Median serum NO levels were 109.79, 170.05, and 227.95 µmol/L, respectively, and median serum MPO levels were 4.14, 8.00, and 9.98 ng/mL, respectively (both *p* < 0.001). GOLD stage 3–4 disease was associated with higher levels of both biomarkers than GOLD stage 1–2 disease (both *p* < 0.001). In COPD-only exploratory logistic regression, serum NO and MPO were associated with AECOPD status. ROC analysis showed AUC values of 0.911 for serum NO and 0.811 for serum MPO, with no significant AUC difference by DeLong’s test (*p* = 0.187). *Conclusions:* Serum MPO and NO levels were progressively higher across healthy control, stable COPD, and AECOPD states. These exploratory findings suggest that systemic oxidative and NO-associated inflammatory activity may reflect COPD clinical state; however, sample-specific ROC cut-offs require external validation before diagnostic use.

## 1. Introduction

Chronic obstructive pulmonary disease (COPD) is a heterogeneous and progressive respiratory disorder characterized by chronic respiratory symptoms and persistent, often progressive airflow obstruction due to abnormalities of the airways and/or alveoli [1]. Beyond spirometric airflow limitation, COPD is increasingly understood as a multidimensional disease in which genetic susceptibility, environmental exposures, airway remodeling, parenchymal destruction, persistent inflammation, systemic inflammatory activity, symptom burden, and exacerbation susceptibility interact across the disease course [1,2]. Acute exacerbations of COPD (AECOPD) are clinically important events because they are associated with accelerated lung function decline, impaired health-related quality of life, increased healthcare utilization, and increased mortality risk [3,4,5,6].

Inflammatory activity in COPD differs according to clinical state. Stable COPD may be accompanied by persistent airway and systemic inflammation, whereas AECOPD represents an acute amplification of inflammatory burden, often associated with increased respiratory symptoms, impaired pulmonary function, infection-related triggers, and increased future exacerbation risk [6,7,8,9]. Neutrophilic inflammation, oxidative stress, and nitrosative inflammatory pathways are central components of COPD pathobiology and may contribute to airway injury, mucus hypersecretion, protease–antiprotease imbalance, tissue remodeling, endothelial dysfunction, and progression of airflow limitation [10,11,12]. Accordingly, circulating biomarkers that reflect systemic oxidative and inflammatory activity may provide complementary biological information beyond spirometric impairment alone.

Myeloperoxidase (MPO) is a neutrophil-associated heme peroxidase released during inflammatory activation and contributes to the generation of reactive oxidant species involved in host defense and inflammatory tissue injury [13]. In COPD, serum MPO has been associated with accelerated decline in forced expiratory volume in one second and adverse cardiovascular outcomes, supporting its potential relevance as a systemic marker of neutrophil-related oxidative activity [14]. However, MPO is not disease-specific and should be interpreted in relation to clinical status, smoking exposure, systemic inflammatory burden, and pulmonary function.

Nitric oxide (NO) is a biologically active mediator involved in airway inflammation, host defense, endothelial regulation, vascular tone, and oxidative inflammatory balance [15]. Altered NO biology has been described in COPD, including changes in nitric oxide synthase activity and endothelial NO pathway dysregulation, suggesting that NO may reflect airway and systemic inflammatory processes in selected COPD phenotypes [16,17]. When assessed together with oxidative biomarkers such as MPO, serum NO may provide additional insight into the systemic inflammatory profile of COPD across different clinical states.

Despite growing interest in systemic biomarkers in COPD, the combined assessment of serum MPO and serum NO across healthy controls, stable COPD, and AECOPD remains insufficiently characterized. Clarifying whether these biomarkers differ across clinical states and whether they show exploratory discriminatory performance for AECOPD may improve the biological characterization of exacerbation-associated systemic inflammation. Therefore, this study aimed to compare serum MPO and serum NO levels among patients with AECOPD, patients with stable COPD, and healthy adult controls. Secondary exploratory analyses evaluated biomarker differences according to GOLD spirometric severity, associations with clinical and spirometric variables, and the discriminatory performance of serum MPO and serum NO for distinguishing AECOPD from stable COPD.

## 2. Materials and Methods

### 2.1. Study Design and Setting

This cross-sectional comparative observational biomarker study with three independent groups was conducted at the Department of Chest Diseases, Harran University Faculty of Medicine, between 20 December 2025 and 20 March 2026. The study was designed to compare systemic oxidative and NO biomarker profiles among patients with AECOPD, patients with stable COPD, and healthy adult controls.

### 2.2. Participant Selection and Study Groups

**Figure 1:**
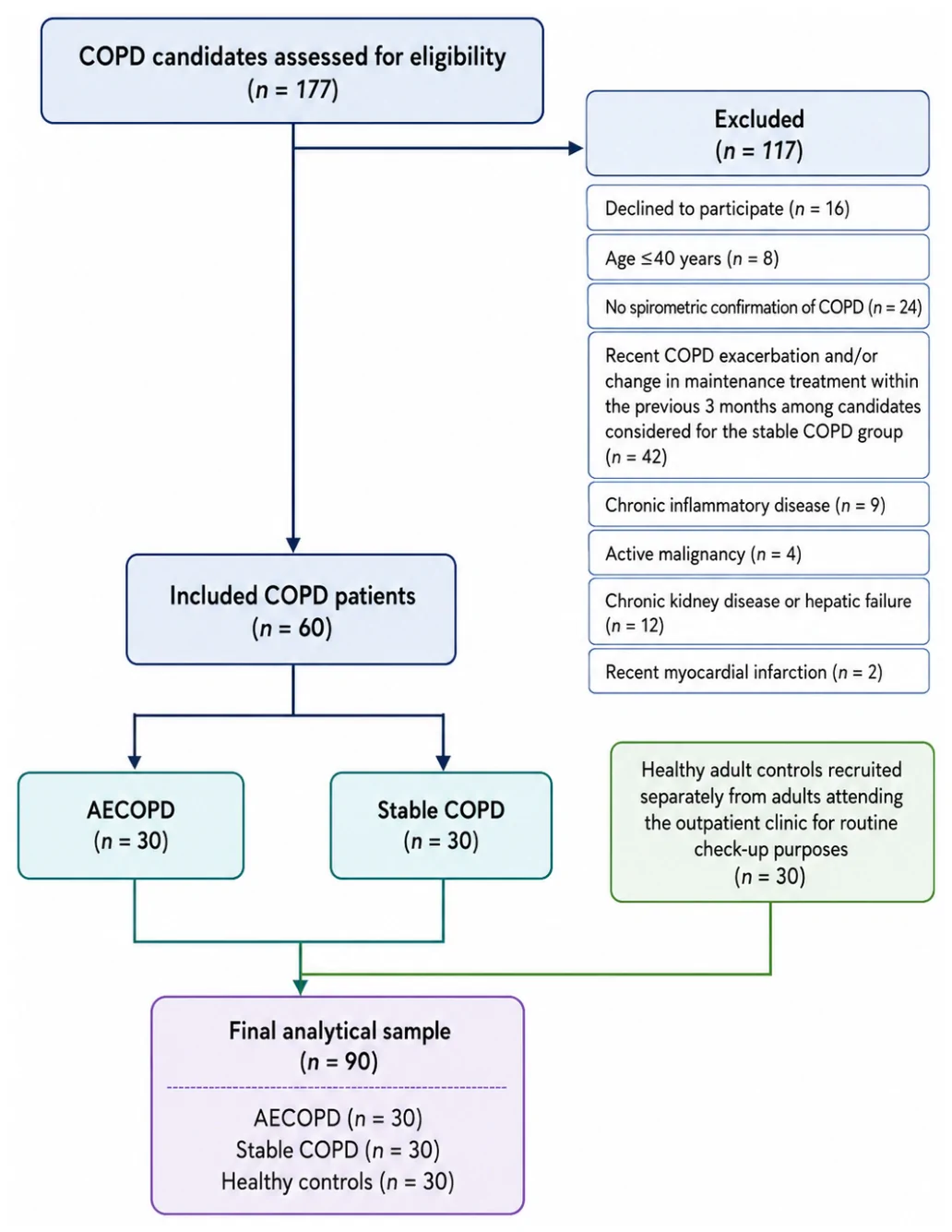
Participant flow diagram. The flow diagram shows the selection of COPD candidates, exclusion of ineligible participants, allocation of included COPD patients into the AECOPD and stable COPD groups, separate recruitment of healthy adult controls from adults attending the outpatient clinic for routine check-up purposes, and the final analytical sample. AECOPD: acute exacerbation of chronic obstructive pulmonary disease; COPD: chronic obstructive pulmonary disease.

COPD candidates were screened for eligibility during the study period. A total of 177 COPD candidates were assessed, of whom 117 were excluded for the following reasons: declined to participate (*n* = 16), age ≤ 40 years (*n* = 8), absence of spirometric confirmation of COPD (*n* = 24), recent COPD exacerbation and/or change in maintenance treatment within the previous three months among candidates considered for the stable COPD group (*n* = 42), chronic inflammatory disease (*n* = 9), active malignancy (*n* = 4), chronic kidney disease or hepatic failure (*n* = 12), and recent myocardial infarction (*n* = 2). Finally, 60 COPD patients were included: 30 patients with AECOPD and 30 patients with stable COPD. The participant selection process is summarized in Figure 1.

An additional control group of 30 healthy adult participants was recruited from adults who presented to the outpatient clinic for routine check-up purposes during the same study period. Controls had no known history of chronic respiratory disease, no acute respiratory symptoms at enrollment, and normal spirometric findings. Age, sex, smoking status, and smoking exposure were recorded and presented as baseline characteristics to characterize group comparability and to support the interpretation of biomarker differences. The final analytical sample included 90 participants: AECOPD (*n* = 30), stable COPD (*n* = 30), and healthy controls (*n* = 30). Control participants were not individually matched to COPD patients; therefore, demographic, smoking-related, and comorbidity differences were reported and considered when interpreting biomarker comparisons.

### 2.3. Diagnostic Definitions

COPD was confirmed by post-bronchodilator spirometry, defined as a forced expiratory volume in one second/forced vital capacity ratio (FEV_1_/FVC) of <0.70, in accordance with the Global Initiative for Chronic Obstructive Lung Disease (GOLD) 2025 Report [18]. AECOPD was defined as an acute worsening of respiratory symptoms requiring additional systemic treatment, and stable COPD was defined as no change in maintenance treatment and no exacerbation during the preceding three months, in accordance with the GOLD 2025 Report [18]. COPD patients were categorized according to the GOLD 2025 ABE assessment system and GOLD spirometric severity [18]. For exploratory subgroup analysis, GOLD spirometric stages were collapsed into GOLD 1–2 and GOLD 3–4 groups.

### 2.4. Inclusion and Exclusion Criteria

Participants in the COPD groups were eligible if they were older than 40 years and had spirometrically confirmed COPD. Exclusion criteria were the presence of other chronic inflammatory diseases, active malignancy, chronic kidney disease, hepatic failure, or a recent history of myocardial infarction. Healthy controls were adults who attended the outpatient clinic for routine check-up purposes, had no known chronic respiratory disease, had no acute respiratory symptoms at enrollment, and had normal spirometric findings. The same major exclusion criteria were applied to control participants where applicable.

### 2.5. Clinical and Spirometric Assessment

Demographic characteristics (age and sex), smoking status (never, current, or former smoker), cumulative smoking exposure (pack-years), the presence of any comorbidity, and the recorded principal comorbidity category were documented at enrollment. The comorbidity variable was coded as absent or present; when present, the source dataset contained one mutually exclusive principal category (hypertension, diabetes mellitus, or cardiovascular disease). Exacerbation history, inhaler treatment, and inhaled corticosteroid (ICS) use were also recorded. COPD symptom burden was assessed using the modified Medical Research Council (mMRC) dyspnea scale and the COPD Assessment Test (CAT) in COPD patients.

Spirometric parameters included FEV1, FEV1% predicted, forced vital capacity (FVC), FVC% predicted, and FEV1/FVC ratio. Spirometric assessments were performed as part of routine pulmonary function testing using a calibrated Quark PFT system (COSMED S.r.l., Albano Laziale, Italy) in the pulmonary function laboratory. Quality control, calibration verification, and acceptability of spirometric maneuvers were assessed according to the routine laboratory protocol. Post-bronchodilator spirometry was used for COPD confirmation, and COPD was diagnosed based on post-bronchodilator FEV1/FVC < 0.70.

### 2.6. Blood Sampling and Serum Preparation

Venous blood samples were collected upon admission in patients with AECOPD and during routine follow-up in patients with stable COPD and healthy controls. In patients with AECOPD, samples were obtained before initiation of additional systemic treatment. In accordance with the ELISA kit instructions, blood samples were collected into non-anticoagulated tubes, allowed to clot at room temperature for 10–20 min, and centrifuged at 3000 rpm for 20 min. The separated serum was aliquoted and stored at −80 °C until biochemical analysis, avoiding repeated freeze–thaw cycles.

### 2.7. Biomarker Measurements

Serum MPO and NO levels were measured using commercially available Human MPO ELISA Kit and Human NO ELISA Kit, respectively (SunLong Biotech Co., Ltd., Hangzhou, China; catalog numbers: SL1230Hu for MPO and SL1275Hu for NO), according to the manufacturer’s instructions. Both assays were based on antibody-coated microplate wells, HRP-labeled antibody conjugate, TMB substrate reaction, acid stop solution, and absorbance measurement at 450 nm. Serum MPO concentrations were reported in ng/mL, with a manufacturer-reported assay range of 0.2–10 ng/mL and sensitivity of 0.05 ng/mL, whereas serum NO concentrations were reported in µmol/L, with an assay range of 10–200 µmol/L and sensitivity of 2.5 µmol/L. The reported intra-assay and inter-assay coefficients of variation were <10% and <12% for both assays. Samples with optical density values exceeding the highest standard were diluted using the manufacturer-recommended sample diluent, re-assayed according to the kit protocol, and final concentrations were calculated by multiplying the measured value by the total dilution factor. Serum NO concentrations were measured using the manufacturer-labeled Human NO ELISA kit and reported according to the manufacturer’s assay specifications. Because of the extremely short biological half-life of nitric oxide, serum NO concentrations measured using the commercial ELISA kit should be interpreted as indirect estimates of systemic nitric oxide production rather than direct measurements of free nitric oxide.

### 2.8. Statistical Analysis

Statistical analyses were performed using IBM SPSS Statistics for Windows, version 27.0 (IBM Corp., Armonk, NY, USA), jamovi version 2.6.44 (The jamovi project, Sydney, Australia) and R version 4.6.0. Continuous variables were evaluated using Shapiro–Wilk tests and Q–Q plots and were summarized as mean ± standard deviation or median with interquartile range, as appropriate; categorical variables were summarized as frequencies and percentages. Age and smoking pack-years were compared across the three study groups using the Kruskal–Wallis test. A significant omnibus result was followed by Dunn–Bonferroni pairwise comparisons. Sex, smoking status, and the presence of any comorbidity were compared using Pearson’s chi-square test. Significant categorical omnibus comparisons were followed by pairwise Fisher’s exact tests for 2 × 2 tables or Fisher–Freeman–Halton exact tests for 2 × 3 tables, with Holm adjustment for the three pairwise group comparisons. Principal comorbidity categories were compared between the AECOPD and stable COPD groups only among participants with a recorded comorbidity. Other group comparisons were performed using one-way analysis of variance, Kruskal–Wallis test with Dunn–Bonferroni post hoc comparisons, independent-samples *t*-test, or Mann–Whitney U test, as appropriate. Effect sizes were reported as eta-squared (η^2^) for one-way analysis of variance, epsilon-squared (ε^2^) for Kruskal–Wallis tests, absolute rank-biserial correlation (|r_rb_|) for Mann–Whitney U tests, and Cramér’s V for categorical comparisons. Spearman’s rank correlation analysis was used to evaluate associations between serum biomarkers and clinical or spirometric variables. Heatmap visualizations of the correlation analyses were generated using the *ggstatsplot* package in R (version 4.6.0) [19]. Exploratory multiple linear regression models were used to assess variables associated with serum MPO and serum NO levels. Binary logistic regression was performed among COPD patients to evaluate the association of serum MPO and NO levels with AECOPD after adjusting for age, sex, smoking pack-years, and forced vital capacity (FVC). Stable COPD and AECOPD were coded as 0 and 1, respectively. Receiver operating characteristic curve analysis was used to evaluate the discriminatory performance of serum MPO and NO for distinguishing AECOPD from stable COPD; Youden’s index was used to determine sample-specific cut-off values, and AUCs were compared using DeLong’s test. A combined NO + MPO ROC model was also evaluated. Bootstrap internal validation with 1000 resamples was performed, and percentile-based 95% confidence intervals were calculated for the AUC estimates. Analyses were performed using available cases, all tests were two-sided, and *p* < 0.05 was considered statistically significant. No formal a priori sample size calculation was performed; therefore, the study was considered exploratory. Except for the specified Dunn–Bonferroni and Holm-adjusted pairwise comparisons, no additional formal multiplicity correction was applied to subgroup, correlation, regression, or ROC analyses, which were interpreted as hypothesis-generating.

### 2.9. Ethical Considerations

The study was approved by the Harran University Clinical Research Ethics Committee (Approval No: HRÜ/25.20.12; Date: 15 December 2025). The study was conducted in accordance with the principles of the Declaration of Helsinki. Written informed consent was obtained from all participants before enrollment.

## 3. Results

### 3.1. Study Population and Baseline Characteristics

A total of 90 participants were included in the final analytical sample, consisting of 30 patients with AECOPD, 30 patients with stable COPD, and 30 healthy controls recruited from adults attending the outpatient clinic for routine check-up purposes. The participant selection process is shown in Figure 1.

Overall, 63 participants (70.0%) were male and 27 (30.0%) were female. In the total cohort, 42 participants (46.7%) were current smokers, 29 (32.2%) were former smokers, and 19 (21.1%) were never-smokers; 44 participants (48.9%) had a recorded comorbidity. Among COPD patients (*n* = 60), 55 patients (91.7%) had experienced at least one exacerbation during the previous year. According to the GOLD 2025 ABE assessment system, 53 COPD patients (88.3%) were classified as GOLD Group E. Spirometric severity distribution among COPD patients was mild in 3 patients (5.0%), moderate in 19 (31.7%), severe in 18 (30.0%), and very severe in 20 (33.3%). Overall, 55 COPD patients (91.7%) were inhaled corticosteroid users.

Age did not differ significantly among the three groups (H = 5.725, *p* = 0.057, ε^2^ = 0.043). Sex distribution differed overall (χ^2^(2) = 20.000, *p* < 0.001, Cramér’s V = 0.471), as did smoking status (χ^2^(4) = 38.307, *p* < 0.001, Cramér’s V = 0.461). Pairwise analyses showed no significant difference between the AECOPD and stable COPD groups in sex (Holm-adjusted *p* = 0.472) or smoking status (Holm-adjusted *p* = 0.132); each COPD group differed from healthy controls after multiplicity adjustment (all adjusted *p* ≤ 0.007). Smoking exposure differed across groups (H = 27.748, *p* < 0.001, ε^2^ = 0.379): pack-years were higher in both COPD groups than in healthy controls (both Dunn–Bonferroni *p* < 0.001), whereas AECOPD and stable COPD did not differ (*p* = 0.275). Any comorbidity was present in 22/30 (73.3%) participants in each COPD group and in none of the healthy controls (χ^2^(2) = 43.043, *p* < 0.001, Cramér’s V = 0.692). The distribution of the recorded principal comorbidity category did not differ between comorbidity-positive AECOPD and stable COPD participants (χ^2^(2) = 0.364, *p* = 0.834, Cramér’s V = 0.091).

Patients with AECOPD had higher systemic inflammatory marker levels, higher serum NO and MPO levels, and more impaired pulmonary function than patients with stable COPD and healthy controls. Baseline demographic, smoking, comorbidity, clinical, laboratory, pulmonary function, and biomarker characteristics are summarized in Table 1.

### 3.2. Serum NO and MPO Levels Across Study Groups

Serum NO and MPO concentrations reported in the Results represent final calculated concentrations after protocol-based dilution and re-assay procedures where applicable. Serum NO and MPO levels differed significantly among the three study groups.

Serum NO levels showed a stepwise increase from healthy controls to stable COPD and AECOPD. The Kruskal–Wallis test showed a significant overall group difference for serum NO levels (χ^2^ = 72.72, df = 2, *p* < 0.001, ε^2^ = 0.82). Dunn–Bonferroni post hoc analysis showed significant differences between all pairwise group comparisons (all *p* < 0.001).

Serum MPO levels showed a similar stepwise pattern, with the highest levels in AECOPD and the lowest levels in healthy controls. The overall group difference was significant for serum MPO (χ^2^ = 66.91, df = 2, *p* < 0.001, ε^2^ = 0.75), and all pairwise comparisons were statistically significant after Dunn–Bonferroni correction (all *p* < 0.001). These results are summarized in Table 2.

### 3.3. Biomarker Levels According to GOLD Severity

In exploratory subgroup analyses, patients with GOLD stage 3–4 COPD had significantly higher serum NO and MPO levels than patients with GOLD stage 1–2 COPD. For serum NO levels, the mean rank was 19.09 in GOLD stage 1–2 and 37.11 in GOLD stage 3–4 (U = 167.0, Z = −3.851, *p* < 0.001). For serum MPO levels, the corresponding mean ranks were 19.16 and 37.07, respectively (U = 168.5, Z = −3.829, *p* < 0.001) Subgroup analyses are presented in Table 3.

### 3.4. Correlation Analyses

**Figure 2:**
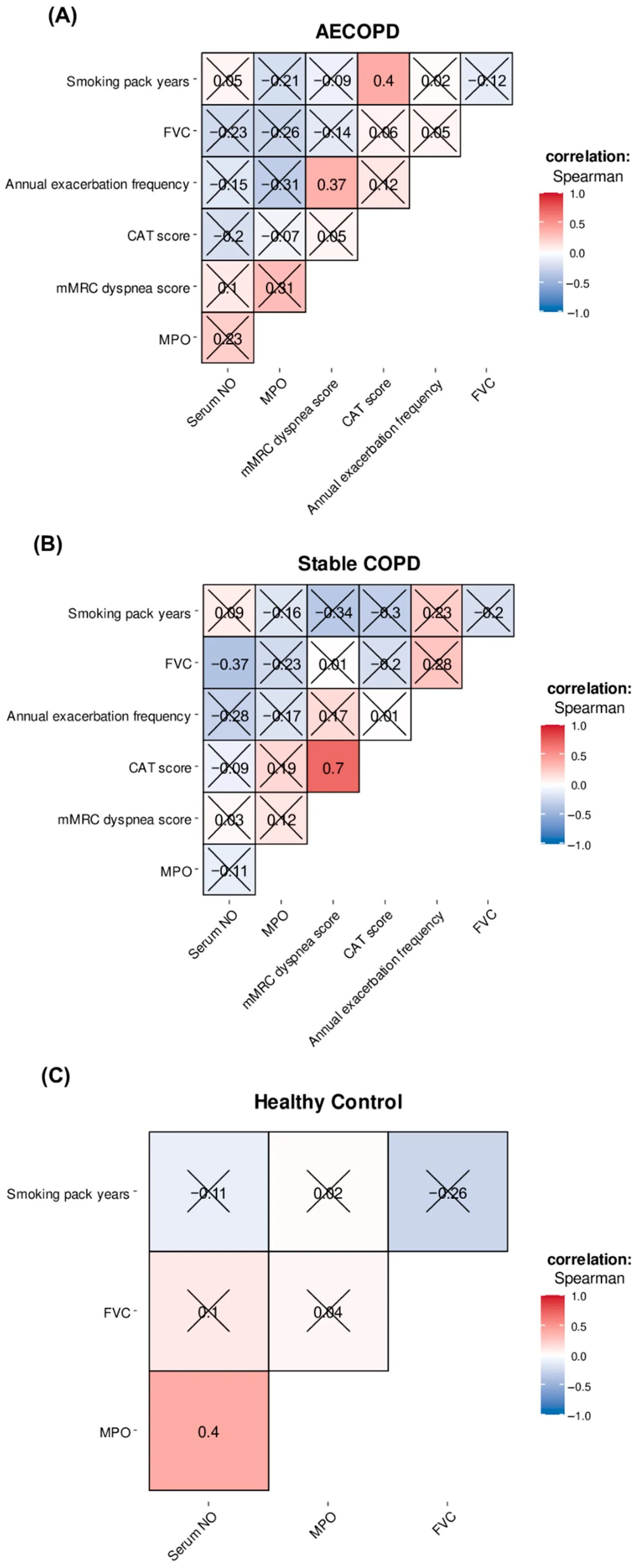
Heatmaps of Spearman correlation coefficients in (**A**) AECOPD, (**B**) stable COPD, and (**C**) healthy control groups. Colors indicate the direction and strength of the Spearman correlation coefficient (ρ), with warmer colors representing positive correlations and cooler colors representing negative correlations. Cells marked with an “X” indicate non-significant correlations (*p* ≥ 0.05).

Selected Spearman correlations are presented in Table 4, and their corresponding heatmaps are shown in Figure 2. In healthy controls, serum NO levels were positively correlated with serum MPO levels (ρ = 0.400, *p* = 0.029). In stable COPD, serum NO levels were inversely correlated with FVC (ρ = −0.365, *p* = 0.047), and mMRC dyspnea score was positively correlated with CAT score (ρ = 0.697, *p* < 0.001). In the AECOPD group, mMRC dyspnea score was positively correlated with annual exacerbation frequency (ρ = 0.367, *p* = 0.046), whereas CAT score was positively correlated with smoking pack-years (ρ = 0.403, *p* = 0.027). No significant Spearman correlation was observed between serum NO or MPO levels and FEV1% predicted within individual study groups.

### 3.5. Exploratory Multiple Linear Regression Analyses

Exploratory multiple linear regression analyses were performed to evaluate variables associated with serum MPO and serum NO levels. Disease status was entered as an ordered group variable reflecting healthy control, stable COPD, and AECOPD status.

In the model with serum MPO as the dependent variable, FEV1 (β = −0.288, *p* = 0.022) and disease status (β = 0.344, *p* = 0.037) were associated with serum MPO levels. Smoking pack-years was not significantly associated with serum MPO levels (*p* = 0.777), whereas serum NO showed borderline statistical significance (β = 0.257, *p* = 0.054).

In the model with serum NO as the dependent variable, disease status (β = 0.427, *p* < 0.001) and serum MPO levels (β = 0.590, *p* < 0.001) were associated with serum NO levels, whereas FEV1% predicted was not statistically significant (*p* = 0.344). The regression models are summarized in Table 5.

### 3.6. Multivariable Binary Logistic Regression Analysis

A multivariable binary logistic regression model was constructed, including forced vital capacity (FVC), age, sex, and smoking pack-years as covariates. Healthy controls were not included in this analysis. Stable COPD and AECOPD were coded as 0 and 1, respectively.

After adjustment for age, sex, smoking pack-years, and FVC, higher serum MPO levels remained independently associated with AECOPD (adjusted OR = 3.150, 95% CI: 1.419–6.993, *p* = 0.005). Higher serum NO levels were also independently associated with increased odds of AECOPD (adjusted OR = 1.101, 95% CI: 1.036–1.170, *p* = 0.002). Logistic regression results are presented in Table 6.

### 3.7. Exploratory ROC Curve Analyses

ROC curve analysis was performed to explore the discriminatory performance of serum NO and MPO levels for distinguishing AECOPD from stable COPD. Healthy controls were not included in the ROC analyses. AECOPD was defined as the positive class and stable COPD as the comparator group.

Serum NO levels showed high apparent discriminatory performance with an AUC of 0.911 (95% CI: 0.835–0.987, *p* < 0.001). The sample-specific cut-off value determined by Youden’s index was 188.47 µmol/L, yielding 86.67% sensitivity and 86.67% specificity. Serum MPO levels also showed significant discriminatory performance, with an AUC of 0.811 (95% CI: 0.695–0.927, *p* < 0.001). The sample-specific cut-off value for MPO was 8.67 ng/mL, corresponding to 76.67% sensitivity and 80.00% specificity.

To evaluate whether combining both biomarkers improved diagnostic performance, a combined ROC model incorporating serum NO and MPO was constructed. The combined model demonstrated the highest discriminatory performance, with an AUC of 0.940 (95% CI: 0.883–0.997, *p* < 0.001), a sample-specific cut-off value of 0.41, 83.33% sensitivity, and 93.33% specificity. DeLong’s test indicated a significant improvement in discriminatory performance for the combined model (*p* = 0.041).

**Figure 3:**
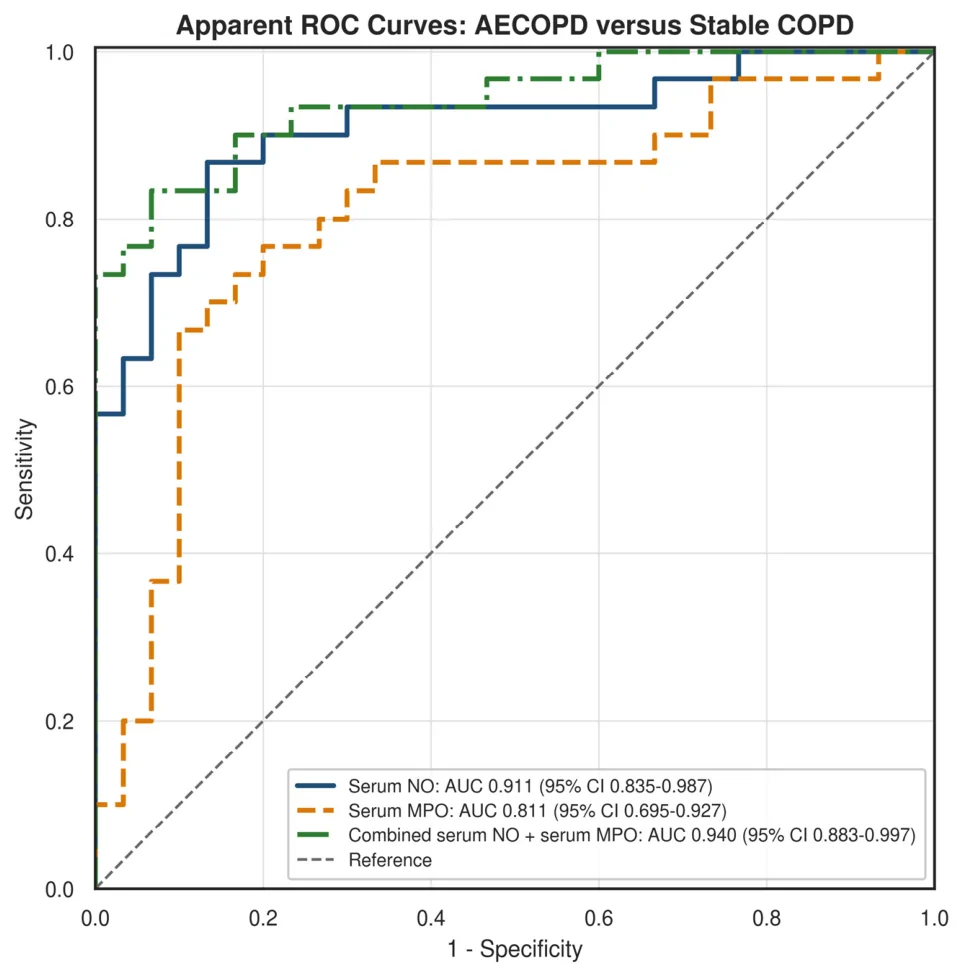
Receiver operating characteristic (ROC) curves for serum nitric oxide (NO), serum myeloperoxidase (MPO), and the combined NO + MPO model in distinguishing acute exacerbation of chronic obstructive pulmonary disease (AECOPD) from stable COPD. The combined model demonstrated the highest discriminatory performance (AUC = 0.940, 95% CI: 0.883–0.997), followed by serum NO (AUC = 0.911, 95% CI: 0.835–0.987) and serum MPO (AUC = 0.811, 95% CI: 0.695–0.927). Sample-specific cut-off values were determined using Youden’s index. DeLong’s test indicated a significant improvement in discriminatory performance for the combined model (*p* = 0.041).

DeLong’s test showed that the difference between the AUC values of serum NO and MPO was not statistically significant (AUC difference = 0.10, 95% CI: −0.05 to 0.25; *z* = 1.32, *p* = 0.187). ROC results are presented in Table 7, and the corresponding ROC curves are shown in Figure 3.

### 3.8. Comparison of Biomarker Discriminatory Performance

To benchmark the diagnostic performance of serum NO and MPO against routinely available inflammatory biomarkers, DeLong’s test was performed to compare the ROC curves of NO and MPO with those of CRP and NLR. Serum NO demonstrated significantly higher discriminatory performance than CRP (AUC difference = 0.30, 95% CI: 0.14–0.46, *p* < 0.001) and NLR (AUC difference = 0.33, 95% CI: 0.17–0.48, *p* < 0.001). Similarly, serum MPO outperformed CRP (AUC difference = 0.20, 95% CI: 0.02–0.37, *p* = 0.026) and NLR (AUC difference = 0.23, 95% CI: 0.04–0.41, *p* = 0.016). These findings indicate that both serum NO and MPO provided superior diagnostic discrimination for distinguishing AECOPD from stable COPD compared with CRP and NLR (Table 8).

### 3.9. Bootstrap Internal Validation

To assess the internal validity of the ROC analyses and evaluate the robustness of the observed diagnostic performance, bootstrap internal validation with 1000 resamples was performed. The bootstrap 95% percentile confidence intervals for the AUC were 0.827–0.979 for serum NO, 0.689–0.921 for serum MPO, and 0.880–0.996 for the combined NO + MPO model. These intervals were broadly consistent with the original ROC estimates, supporting the stability of the observed diagnostic performance across bootstrap resamples. Bootstrap validation results are summarized in Table 9.

## 4. Discussion

In this cross-sectional comparative observational biomarker study, serum MPO and NO levels differed significantly among healthy controls, patients with stable COPD, and patients with AECOPD. Both biomarkers showed a stepwise increase from healthy controls to stable COPD and reached their highest levels during AECOPD. In COPD-only exploratory analyses, serum MPO and NO levels were higher in patients with GOLD stage 3–4 disease than in those with GOLD stage 1–2 disease. Exploratory regression analyses also showed that disease status was associated with both serum MPO and serum NO levels. In ROC analyses, both biomarkers showed apparent discriminatory performance for distinguishing AECOPD from stable COPD; however, the derived cut-off values should be interpreted as sample-specific and hypothesis-generating rather than clinically validated diagnostic thresholds. Combining serum NO and MPO further improved the discriminatory performance for distinguishing AECOPD from stable COPD compared with either biomarker alone. Although this combined model remains exploratory and requires external validation, the findings suggest that integrating biomarkers reflecting complementary biological pathways, including oxidative stress and nitric oxide-related inflammatory signaling, may provide greater diagnostic value than reliance on a single biomarker. These findings are consistent with previous reviews emphasizing the potential value of complementary oxidative stress biomarkers and the need for further validation in COPD [12].

The observed increase in serum MPO across the COPD clinical spectrum is consistent with the contribution of neutrophilic inflammation and oxidative stress to COPD pathobiology. COPD is increasingly recognized as a heterogeneous disease in which airway inflammation, systemic inflammatory activity, oxidative stress, and exacerbation susceptibility interact across the disease course [1,2]. MPO is a neutrophil-associated heme peroxidase released during inflammatory activation and contributes to reactive oxidant generation [16]. Previous studies have shown that oxidative stress is present in stable COPD and becomes more pronounced during exacerbations [9,10,11,12,13,14,15]. Therefore, the highest MPO levels observed in AECOPD may reflect exacerbation-associated amplification of neutrophil-related oxidative activity. This interpretation is supported by evidence from Park et al., who reported that circulating MPO was associated with accelerated FEV1 decline and adverse outcomes in COPD [17].

Serum NO levels also increased progressively from healthy controls to stable COPD and AECOPD. NO is a biologically active mediator involved in airway inflammation, host defense, vascular regulation, endothelial function, and redox balance in the respiratory system [20]. Altered NO biology has been reported in COPD, including changes in nitric oxide synthase activity in peripheral lung tissue and dysregulation of endothelial NO-related pathways [21,22]. In the present study, higher serum NO levels in COPD, particularly during AECOPD, may therefore reflect increased systemic inflammatory and vascular-biological activity. The parallel increase in serum NO and MPO suggests that oxidative and NO-associated inflammatory pathways may coexist during COPD progression and exacerbation-related inflammatory activation. Nevertheless, serum NO should be interpreted as a systemic biomarker signal rather than as a disease-specific indicator of COPD.

The higher serum MPO and NO levels observed in GOLD stage 3–4 disease suggest that systemic oxidative and NO-associated inflammatory activity may be more pronounced in advanced airflow limitation. However, neither biomarker showed significant within-group correlations with FEV1% predicted. This finding is clinically plausible because COPD is biologically heterogeneous, and single circulating biomarkers may distinguish broad clinical states without demonstrating strong linear relationships with spirometric indices at the individual-patient level [1,2,6,7,8]. Accordingly, serum MPO and NO should not be interpreted as substitutes for spirometric assessment. Rather, they may reflect systemic inflammatory dimensions that complement physiological evaluation.

The exploratory regression analyses provide additional biological context. Disease status was associated with both serum MPO and serum NO levels, supporting the observed biomarker gradient across healthy controls, stable COPD, and AECOPD. FEV1 was associated with serum MPO in the MPO model, whereas FEV1% predicted was not significantly associated with serum NO in the NO model. These findings may suggest that MPO is more closely related to airflow limitation-associated oxidative burden, whereas serum NO may reflect broader inflammatory or endothelial-biological processes. However, the regression models were exploratory, and disease status was entered as an ordered group variable; therefore, these findings should be considered hypothesis-generating rather than causal.

The COPD-only logistic regression and ROC analyses suggested that serum MPO and NO may have exploratory discriminatory value for distinguishing AECOPD from stable COPD. Serum NO showed a numerically higher AUC than serum MPO, but DeLong’s test did not demonstrate a statistically significant difference between the two AUCs. This pattern suggests that serum NO and MPO may provide partially complementary biological information. Importantly, the ROC-derived thresholds were obtained from the present cohort and were not externally validated. Therefore, these cut-off values should not be considered clinically generalizable diagnostic thresholds. This distinction is essential because diagnostic accuracy estimates from relatively small single-center biomarker studies may be optimistic and require independent validation before clinical translation [23]. In addition, both serum NO and MPO demonstrated significantly greater discriminatory performance than the routinely available inflammatory biomarkers CRP and NLR. Although CRP and NLR remain inexpensive and widely used inflammatory markers, their diagnostic specificity for COPD exacerbations is limited because they primarily reflect systemic inflammation rather than oxidative or nitrosative stress. The superior performance of NO and MPO observed in our cohort suggests that these biomarkers may better reflect biological processes directly involved in COPD exacerbation and may provide complementary diagnostic information beyond conventional inflammatory markers [12,24].

Several limitations should be acknowledged. First, this was a single-center study with a modest sample size, which limits generalizability and statistical power, particularly for subgroup analyses. Second, biomarker measurements were obtained at a single time point, preventing assessment of longitudinal changes during exacerbation recovery or stable follow-up. Third, the etiology and inflammatory phenotype of exacerbations were not systematically classified; therefore, infectious, eosinophilic, or other biological exacerbation phenotypes could not be evaluated separately. Fourth, dietary factors, endothelial comorbidities, renal handling, and other potential determinants of serum NO were not comprehensively controlled. Fifth, healthy controls were recruited separately from routine check-up attendees and were not individually matched to COPD patients; baseline sex, smoking-status, and comorbidity distributions differed between COPD and control groups, and residual confounding in unadjusted group comparisons cannot be excluded. Sixth, detailed assay-level information such as lot-specific validation, duplicate measurement records, technician blinding, batch effects, and hemolysis/lipemia/icterus indices was not available for all samples, which should be considered when interpreting assay-level variability. Finally, although bootstrap internal validation supported the stability of the observed ROC estimates, the diagnostic models remain exploratory and lacked calibration assessment, decision-curve analysis, and external validation. Therefore, the reported thresholds should be considered preliminary and sample-specific.

## 5. Conclusions

Serum MPO and NO levels were progressively higher across healthy control, stable COPD, and AECOPD states, suggesting that systemic oxidative and NO-associated inflammatory activity may differ according to COPD clinical state. Higher biomarker levels in GOLD stage 3–4 disease support an association with more advanced airflow limitation, but these findings should not be interpreted causally. Serum MPO and NO showed exploratory discriminatory performance for distinguishing AECOPD from stable COPD; however, the ROC-derived thresholds were sample-specific and require validation in larger independent cohorts before clinical application.

## Figures and Tables

**Table 1:** Demographic, Smoking, Comorbidity, Clinical, Laboratory, Pulmonary Function, and Biomarker Characteristics of the Study Groups.

Variable	AECOPD (*n* = 30)	Stable COPD (*n* = 30)	Healthy Controls (*n* = 30)	*p*-Value	Effect Size
Age, years	68.00 (13.75)	66.50 (14.75)	58.00 (16.75)	0.057	ε^2^ = 0.043
Male sex, n (%) ^d^	27 (90.0)	24 (80.0)	12 (40.0)	<0.001	V = 0.471
Smoking status, n (%) ^d^				<0.001	V = 0.461
Never-smoker	2 (6.7)	0 (0.0)	17 (56.7)		
Former smoker	10 (33.3)	16 (53.3)	3 (10.0)		
Current smoker	18 (60.0)	14 (46.7)	10 (33.3)		
Smoking, pack-years ^c^	28.00 (9.00)	24.00 (4.00)	15.00 (4.00)	<0.001	ε^2^ = 0.379
Any comorbidity, n (%) ^d^	22 (73.3)	22 (73.3)	0 (0.0)	<0.001	V = 0.692
Principal comorbidity category, n (%) ^e^			—	0.834	V = 0.091
Hypertension	10 (45.5)	12 (54.5)	—		
Diabetes mellitus	6 (27.3)	5 (22.7)	—		
Cardiovascular disease	6 (27.3)	5 (22.7)	—		
CRP, mg/dL	2.19 (3.84)	0.93 (1.53)	0.04 (0.45)	<0.001	ε^2^ = 0.424
WBC, ×10^3^/µL ^a^	12.27 ± 4.39	9.47 ± 2.45	8.46 ± 2.62	<0.001	η^2^ = 0.201
Neutrophil, ×10^3^/µL	8.97 (6.95)	5.35 (3.51)	4.81 (2.47)	<0.001	ε^2^ = 0.200
Lymphocyte, ×10^3^/µL	1.72 (1.27)	1.46 (1.09)	2.45 (1.25)	0.018	ε^2^ = 0.070
Monocyte, ×10^3^/µL	0.69 (0.40)	0.71 (0.36)	0.57 (0.23)	0.263	ε^2^ = 0.008
Eosinophil, ×10^3^/µL	0.15 (0.34)	0.16 (0.26)	0.14 (0.21)	0.426	ε^2^ = 0.000
HGB, g/dL ^a^	14.26 ± 2.15	13.65 ± 2.70	12.93 ± 2.28	0.102	η^2^ = 0.051
HCT, % ^a^	43.78 ± 5.72	42.09 ± 6.91	37.94 ± 6.31	0.002	η^2^ = 0.135
PLT, ×10^3^/µL	258.70 (74.02)	252.00 (82.00)	280.00 (89.18)	0.152	ε^2^ = 0.020
MPV, fL ^a^	9.42 ± 1.53	8.84 ± 1.82	10.32 ± 1.12	0.001	η^2^ = 0.141
NLR	4.26 (6.80)	3.50 (4.48)	1.82 (0.81)	<0.001	ε^2^ = 0.254
PLR	143.39 (100.42)	178.18 (143.00)	124.99 (82.38)	0.166	ε^2^ = 0.018
Urea, mg/dL	37.45 (17.12)	38.52 (27.82)	34.24 (11.77)	0.176	ε^2^ = 0.017
Creatinine, mg/dL	0.92 (0.22)	1.02 (0.28)	0.76 (0.26)	0.008	ε^2^ = 0.089
ALT, U/L	20.50 (33.50)	16.50 (29.00)	17.00 (16.75)	0.287	ε^2^ = 0.006
AST, U/L	21.50 (8.75)	23.50 (13.25)	20.50 (7.75)	0.181	ε^2^ = 0.016
mMRC dyspnea score ^b^	4.00 (1.00)	3.00 (1.00)	—	<0.001	|r_rb_| = 0.492
CAT score ^b^	30.00 (3.75)	26.00 (14.00)	—	0.003	|r_rb_| = 0.444
Exacerbations in previous year ^b^	2.50 (1.00)	1.00 (1.00)	—	<0.001	|r_rb_| = 0.648
FEV1, L	0.83 (0.44)	1.52 (0.77)	3.44 (0.81)	<0.001	ε^2^ = 0.694
FEV1, % predicted	28.00 (9.75)	63.00 (31.25)	91.00 (7.50)	<0.001	ε^2^ = 0.715
FVC, L ^a^	1.96 ± 0.62	2.22 ± 0.81	3.90 ± 0.82	<0.001	η^2^ = 0.574
FVC, % predicted	56.00 (18.50)	73.50 (29.75)	88.50 (10.75)	<0.001	ε^2^ = 0.520
FEV1/FVC, %	41.00 (8.57)	66.50 (10.50)	83.50 (3.63)	<0.001	ε^2^ = 0.834

Data are presented as median (interquartile range), mean ± standard deviation, or n (%), as appropriate. Variables summarized as mean ± standard deviation were compared using one-way analysis of variance; variables summarized as median (interquartile range) were compared using the Kruskal–Wallis test unless otherwise specified. Categorical variables were compared using Pearson’s chi-square test. Significant Kruskal–Wallis comparisons were followed by Dunn–Bonferroni tests; significant categorical comparisons were followed by pairwise Fisher’s exact or Fisher–Freeman–Halton tests with Holm adjustment. ^a^ One-way analysis of variance. ^b^ Mann–Whitney U test between COPD groups only. ^c^ Available sample sizes for smoking pack-years were *n* = 28 for AECOPD, *n* = 30 for stable COPD, and *n* = 13 for healthy controls. ^d^ The *p*-value and Cramér’s V refer to the overall categorical distribution. ^e^ Comorbidity-category percentages were calculated among comorbidity-positive participants (*n* = 22 in each COPD group); healthy controls were not included in this comparison. Effect sizes are reported as η^2^ for one-way analysis of variance, ε^2^ for Kruskal–Wallis tests, |r_rb_| for Mann–Whitney U tests, and Cramér’s V for categorical comparisons. AECOPD: acute exacerbation of chronic obstructive pulmonary disease; ALT: alanine aminotransferase; AST: aspartate aminotransferase; CAT: COPD Assessment Test; COPD: chronic obstructive pulmonary disease; CRP: C-reactive protein; FEV1: forced expiratory volume in one second; FVC: forced vital capacity; HCT: hematocrit; HGB: hemoglobin; mMRC: modified Medical Research Council; MPV: mean platelet volume; NLR: neutrophil-to-lymphocyte ratio; PLR: platelet-to-lymphocyte ratio; PLT: platelet count; WBC: white blood cell count.

**Table 2:** Group Comparisons of Serum NO and MPO Levels.

Biomarker	AECOPD (*n* = 30)	Stable COPD (*n* = 30)	Healthy Controls (*n* = 30)	χ^2^	df	*p*-Value	ε^2^	Dunn–Bonferroni Post Hoc Result
Serum NO, µmol/L	227.95 (49.34)	170.05 (28.29)	109.79 (18.00)	72.72	2	<0.001	0.82	All pairwise comparisons *p* < 0.001
Serum MPO, ng/mL	9.98 (2.53)	8.00 (1.06)	4.14 (2.28)	66.91	2	<0.001	0.75	All pairwise comparisons *p* < 0.001

Data are presented as median (interquartile range). Overall comparisons were performed using the Kruskal–Wallis test. Pairwise comparisons were performed using Dunn–Bonferroni post hoc analysis. AECOPD: acute exacerbation of chronic obstructive pulmonary disease; COPD: chronic obstructive pulmonary disease; MPO: myeloperoxidase; NO: nitric oxide; ε^2^: epsilon-squared effect size.

**Table 3:** Exploratory Subgroup Analyses According to GOLD Severity.

Subgroup Analysis	Biomarker	Groups Compared	Summary Result	Test Statistic	*p*-Value
GOLD severity	Serum NO	GOLD 1–2 vs. GOLD 3–4	Mean rank: 19.09 vs. 37.11	U = 167.0; Z = −3.851	<0.001
GOLD severity	Serum MPO	GOLD 1–2 vs. GOLD 3–4	Mean rank: 19.16 vs. 37.07	U = 168.5; Z = −3.829	<0.001

Data are presented as mean rank or mean ± standard deviation according to the statistical test used. GOLD subgroup comparisons were performed using the Mann–Whitney U test. GOLD: Global Initiative for Chronic Obstructive Lung Disease; MPO: myeloperoxidase; NO: nitric oxide.

**Table 4:** Selected Spearman Correlations Identified Within Study Groups.

Variable Pair	Healthy Controls	Stable COPD	AECOPD
Serum NO–MPO	0.400 (0.029)	−0.113 (0.551)	0.226 (0.230)
Serum NO–FVC	−0.101 (0.597)	−0.365 (0.047)	−0.223 (0.215)
mMRC dyspnea score–CAT score	–	0.697 (<0.001)	0.051 (0.789)
mMRC dyspnea score–annual exacerbation frequency	–	0.174 (0.357)	0.367 (0.046)
CAT score–smoking pack-years	–	−0.301 (0.107)	0.403 (0.027)

Selected biomarker-related and symptom-related correlations considered most relevant to the study objectives are shown. Correlations were assessed using Spearman’s rank correlation analysis and are expressed as Spearman’s correlation coefficient (ρ), with corresponding *p*-values shown in parentheses. Variables without available measurements in healthy controls are indicated by “–”. AECOPD: acute exacerbation of chronic obstructive pulmonary disease; CAT: COPD Assessment Test; FVC: forced vital capacity; mMRC: modified Medical Research Council; MPO: myeloperoxidase; NO: nitric oxide.

**Table 5:** Exploratory Multiple Linear Regression Analyses for Serum MPO and Serum NO Levels.

Dependent Variable	Independent Variable	B	SE	β	*t*	*p*-Value	VIF
MPO	Serum NO	0.013	0.007	0.257	1.959	0.054	2.989
MPO	FEV1, L	−0.793	0.338	−0.288	−2.344	0.022	2.619
MPO	Disease status	1.303	0.613	0.344	2.127	0.037	4.556
MPO	Smoking, pack-years	−0.012	0.041	−0.029	−0.284	0.777	1.836
Serum NO	Disease status	36.473	9.175	0.427	3.975	<0.001	4.235
Serum NO	Serum MPO	11.537	1.562	0.590	7.384	<0.001	2.342
Serum NO	FEV1, % predicted	0.242	0.254	0.097	0.952	0.344	3.770

SE: standard error; β: standardized beta coefficient; VIF: variance inflation factor; FEV1: forced expiratory volume in one second; MPO: myeloperoxidase; NO: nitric oxide. Regression analyses were exploratory. Disease status was entered into the regression models as an ordinal variable and coded as 0 = healthy controls, 1 = stable COPD, and 2 = acute exacerbation of chronic obstructive pulmonary disease (AECOPD).

**Table 6:** Multivariable Logistic Regression Analysis of Factors Associated with AECOPD Among Patients with COPD.

Predictor	B	SE	Wald	*p*-Value	Adjusted OR	95% CI for OR
Serum MPO	1.148	0.407	7.956	0.005	3.150	1.419–6.993
Serum NO	0.096	0.031	9.709	0.002	1.101	1.036–1.170
FVC	1.419	0.727	3.816	0.051	4.134	0.995–17.175
Age	−0.001	0.045	0.001	0.978	0.999	0.914–1.091
Smoking pack-years	0.097	0.117	0.692	0.406	1.102	0.876–1.387
Male sex	−1.100	1.531	0.517	0.472	0.333	0.017–6.687
Constant	−34.167	10.392	10.809	0.001	0.000	—

SE: standard error; OR: odds ratio; CI: confidence interval; FVC: forced vital capacity; MPO: myeloperoxidase; NO: nitric oxide. Regression analyses were exploratory. Stable COPD and AECOPD were coded as 0 and 1, respectively. The model was adjusted for age, sex, smoking pack-years, and FVC.

**Table 7:** Exploratory ROC Analysis of Serum NO, Serum MPO, and the Combined NO + MPO Model for Distinguishing AECOPD from Stable COPD.

Biomarker	AUC (95% CI)	Sample-Specific Cut-Off (95% CI)	Sensitivity (95% CI)	Specificity (95% CI)	LR+	LR−	PPV	NPV	Accuracy
Serum NO	0.911 (0.835–0.987)	188.47 (181.89–212.85)	86.67% (69.28–96.24)	86.67% (69.28–96.24)	6.50	0.15	86.67%	86.67%	86.67%
Serum MPO	0.811 (0.695–0.927)	8.67 (8.21–9.31)	76.67% (57.72–90.07)	80.00% (61.43–92.29)	3.83	0.29	79.31%	77.42%	78.33%
Combined NO + MPO Model	0.940 (0.883–0.997)	0.41 (0.21–0.75)	83.33% (65.28–94.36)	93.33% (77.93–99.18)	12.50	0.18	92.59%	84.85%	88.33%

DeLong’s test comparing serum NO and MPO AUC values: AUC difference = 0.10, 95% CI: −0.05 to 0.25; z = 1.32; *p* = 0.187. DeLong’s test indicated a significant improvement in discriminatory performance for the combined model (*p* = 0.041). AECOPD: acute exacerbation of chronic obstructive pulmonary disease; AUC: area under the curve; CI: confidence interval; COPD: chronic obstructive pulmonary disease; LR+: positive likelihood ratio; LR−: negative likelihood ratio; MPO: myeloperoxidase; NO: nitric oxide; NPV: negative predictive value; PPV: positive predictive value; ROC: receiver operating characteristic. Cut-off values were determined using Youden’s index. Confidence intervals for cut-off values were estimated using bootstrap resampling in R. Cut-off values were derived from the present cohort and should be considered sample-specific.

**Table 8:** DeLong Comparisons Between MPO/NO and Routine Clinical Biomarkers.

Comparison	AUC Difference	95% CI	*p*-Value
NO vs. CRP	0.30	0.14–0.46	<0.001
MPO vs. CRP	0.20	0.02–0.37	0.026
NO vs. NLR	0.33	0.17–0.48	<0.001
MPO vs. NLR	0.23	0.04–0.41	0.016

ROC curve comparisons were performed using DeLong’s nonparametric test. CRP: C-reactive protein; NLR: neutrophil-to-lymphocyte ratio; MPO: myeloperoxidase; NO: nitric oxide.

**Table 9:** Bootstrap Internal Validation of ROC Models (1000 Resamples).

Model	Original AUC (95% CI)	Bootstrap 95% CI (1000 Resamples)
Serum NO	0.911 (0.835–0.987)	0.827–0.979
Serum MPO	0.811 (0.695–0.927)	0.689–0.921
Combined NO + MPO model	0.940 (0.883–0.997)	0.880–0.996

Bootstrap internal validation was performed using 1000 resamples. Bootstrap confidence intervals are percentile-based.

## Data Availability

The data presented in this study are available from the corresponding author upon reasonable request. The data are not publicly available due to privacy and ethical restrictions.
